# Levels of hepatic Th17 cells and regulatory T cells upregulated by hepatic stellate cells in advanced HBV-related liver fibrosis

**DOI:** 10.1186/s12967-017-1167-y

**Published:** 2017-04-11

**Authors:** Xiaoyan Li, Yujie Su, Xuefeng Hua, Chan Xie, Jing Liu, Yuehua Huang, Liang Zhou, Min Zhang, Xu Li, Zhiliang Gao

**Affiliations:** 1 Department of Infectious Diseases, The Third Affiliated Hospital of Sun Yat-sen University, No 600 Tianhe Road, Guangzhou, 510630 Guangdong Province China; 2 Department of Transplant Center, The Third Affiliated Hospital of Sun Yat-sen University, Guangzhou, China; 3 Guangdong Province Key Laboratory of Liver Disease, The Third Affiliated Hospital of Sun Yat-sen University, Guangzhou, China; 4 Department of Infectious Diseases, The First Affiliated Hospital of Anhui Medical University, No 210 Jixi Road, Hefei, 230022 Anhui Province China; 5 Key Laboratory of Tropical Disease Control (Sun Yat-sen University), Ministry of Education, Guangzhou, China

**Keywords:** Th17 cells, Regulatory T cells, Hepatic stellate cells, Liver fibrosis, HCC

## Abstract

**Background:**

Liver fibrosis which mainly occurs upon chronic hepatitis virus infection potentially leads to portal hypertension, hepatic failure and hepatocellular carcinoma. However, the immune status of Th17 and Treg cells in liver fibrosis is controversial and the exact mechanisms remain largely elusive.

**Methods:**

Liver tissues and peripheral blood were obtained simultaneously from 32 hepatitis B virus infected patients undergoing surgery for hepatocellular carcinoma at the medical center of Sun Yat-sen University. Liver tissues at least 3 cm away from the tumor site were used for the analyses. Levels of Th17 cells and regulatory T cells were detected by flow cytometry analysis and immunohistochemistry. In vitro experiment, we adopted magnetic cell sorting to investigate how hepatic stellate cells regulate the levels of Th17 cells and regulatory T cells.

**Results:**

We found that hepatic Th17 cells and regulatory T cells were increased in patients with advanced stage HBV-related liver fibrosis. Hepatic stellate cells upregulated the levels of Th17 cells and regulatory T cells via PGE2/EP2 and EP4 pathway.

**Conclusions:**

We found that the increased levels of Th17 cells and regulatory T cells were upregulated by hepatic stellate cells. These results may provide insight into the role of hepatic stellate cells and Th17 cells and regulatory T cells in the persistence of fibrosis and into the occurrence of hepatocellular carcinoma following cirrhosis.

**Electronic supplementary material:**

The online version of this article (doi:10.1186/s12967-017-1167-y) contains supplementary material, which is available to authorized users.

## Background

Liver fibrosis is the wound-healing response of the liver to many causes of chronic injury, of which hepatitis B virus (HBV) infection is the most common in China [1]. Hepatic stellate cells (HSC) have dominated studies exploring mechanisms of liver fibrosis over the last two decades [2]. Characterizing the interaction of HSC with immune cells is a research priority, yet has been largely overlooked until recently [3]. Moreover, chronic liver injury is associated with varying degrees of hepatic fibrosis, yet its relationship to immune cells status is unknown. In recent studies, we have established that activated HSC from patients suffering from hepatitis B can modulate the phenotype and function of monocytes and NK cells [4–6]. These findings support the hypothesis that HSC from hepatitis B patients plays an important role in regulating the immune status of hepatic fibrosis.

IL17-producing CD4+ T (Th17) cells and regulatory T cells (Tregs) have been recognized as unique subsets of effector T cells that are distinct from the Th1 and Th2 subsets [7–9]. Moreover, they have been implicated as potent effectors of autoimmune disorders and inflammation [10–12]. Particularly, it is found that they were all involved in the pathogenesis of liver fibrosis [13, 14]. It is argued that IL-17 was upregulated in fibrotic liver tissue, which promoted pro-inflammatory cytokine expression, neutrophil influx, liver injury, inflammation, and fibrosis [14–17]. Similarly, the enrichment of Tregs in the livers of patients with advanced fibrosis was found and these Tregs might enhance fibrosis by releasing IL-8 [18]. Both Th17 and Tregs were enriched in tumors or marginal region of HCC and increased intratumoral IL-17-producing cell or Tregs density predicted poor prognosis in HCC patients [19–21]. However, the relationship between Th17 cells, Tregs and different stages of liver fibrosis remains elusive and further investigation is warranted and basically no research available so far to detect the effect of HSC on the status of Th17 cells as well as Tregs in HBV-related liver fibrosis (HBV-LF).

In previous studies, we found that HSC can secrete many kinds of cytokines and chemokines to affect the immune status of hepatic fibrosis, therefore we propose an hypothesis that HSC may also modulate the phenotype and function of Th17 cells and Tregs. To this end, we compared the levels of Th17 cells and CD4+ CD25+ Foxp3+ Tregs in patients with different stages of HBV-LF by flow cytometry and immunohistochemistry. Afterwards, we explored how HSC regulate Th17 cells and Tregs in vitro, attempting to support some available evidences for the future study of mechanisms in fibrosis persistence and hepatocellular carcinoma (HCC) pathogenesis following cirrhosis.

## Methods

### Patients and specimens

Liver tissues and peripheral blood (PB) were obtained simultaneously from 32 HBV-infected patients undergoing surgery for HCC at the medical centre of Sun Yat-sen University. Liver tissues at least 3 cm away from the tumor site were used for the analysis, as described by other investigators [22]. The buffy coats used for in vitro sorting of CD4+ T cells with anti-CD4-labelled magnetic beads were from the blood bank in Guangzhou city in China. All patients were negative for antibodies against hepatitis C virus (HCV), human immunodeficiency virus, and syphilis, in order to avoid their influence on immune status. All samples were coded in accordance with local ethical guidelines as stipulated by the Declaration of Helsinki (Version Fortaleza 2013). Written informed consent was obtained from the patients, and the protocol was approved by the Review Board of Sun Yat-sen University.

### Sirius red staining for liver fibrosis

Staining of hepatic fibrosis was carried out as described in Additional file 1. As mentioned before, liver tissues were evaluated for the presence or absence of bridging fibrosis or cirrhosis by the Laennec system (haematoxylin and eosin stain) [23, 24], and the subjects were categorized accordingly (details in Additional file 1).

### Isolation of mononuclear cells

Peripheral blood mononuclear cells (PBMCs) were isolated from the blood of patients using Ficoll density gradients, as described previously [6]. Fresh tissue mononuclear cells were obtained as previously described [4] (details in Additional file 1). The mononuclear cells were washed and resuspended in medium supplemented with 1% heat-inactivated FCS for flow cytometry analysis.

### Isolation of primary HSC

Isolation of primary HSC (pHSC) were similar with the procedure described previously [25] (details in Additional file 1). They were passaged for 3–8 passage doublings and used for subsequent experiments to minimize the clonal selection and culture stress that may occur during extended tissue culture.

### Immunohistochemistry and immunofluorescence

Paraffin-embedded liver samples were cut into 5 μm sections and processed for immunohistochemistry as previously described [4] (details in Additional file 1). Positive cells were quantified using ImagePro Plus software (Media Cybernetics, America) and detected using microscopy (Leica, Germany).

For immunofluorescence, fibroblasts grown in chamber slides were fixed, pre-incubated with 4% goat serum, and stained with antibodies(details in Additional file 1). Images were acquired using a fluorescence microscope (LEICA DMI 4000B, Germany) and analysed using Leica Application suite software (version 4.0).

### Magnetic cell sorting (MACS)

Purified CD4+ T cells were isolated from the buffy coats derived from the blood of healthy donors using anti-CD4-labelled magnetic beads (MiltenyiBiotec, BergischGladbach, Germany) and then were washed and resuspended in medium supplemented with 1% heat-inactivated FBS for flow cytometry analysis. The cell purity of the CD4+ cells, as assessed by flow cytometry analysis, was greater than 97%.

### In vitro T cell culture system

We plated purified CD4+ T cells in 12-well plates at a concentration of 1 × 10^6^ cells per well per 1 ml RPMI 1640 medium (with 10% heat-inactivated FBS) containing anti-CD3 (2 µg/ml), anti-CD28 (1 µg/ml) and IL-2 (20 U/ml). CD4+ T cells were cultured with 30% supernatants with or without pretreated with 5 µg/µl NS398 (selectiveCOX-2 inhibitor) (Cayman Chemical, USA) from pHSC, LX-2 (an immortal human HSC cell line), or L02 (normal human hepatocytes) (gift from State Key Laboratory of Biocontrol, Guangzhou, China) for 5 days. Next, we cultured purified CD4+ T cells with 0.1 µM Prostaglandin E2 (PGE2), 0.1 µM Butaprost or 0.1 µM Misoprostol (Cayman Chemical, USA) for 5 days. Cells were collected and washed. Flow staining was then performed following the protocols for flow cytometry analysis described above.

### Flow cytometry analysis

Mononuclear cells were isolated from the blood and liver tissues of patients using Ficoll density gradients as described in Additional file 1. These cells were stained for surface markers, fixed, permeabilized and further stained with mAbs directed against intracellular markers (Additional file 1). Data were acquired on a Gallios instrument (Gallios, Beckman Coulter, USA) and analyzed with FlowJo software (TreeStar Inc., Ashland, OR, USA).

### Statistical analysis

Results are expressed as the mean ± SEM. The statistical significance of differences between groups was determined by Student’s t test or ANOVA (testing more than two data sets). We compared two groups using Student’s t test for continuous clinical variables and χ^2^ test for categorical clinical variables. SPSS statistical software (version 13.0) was used for all statistical analysis. All data were analyzed using two-tailed tests unless otherwise specified, and *p* < 0.05 was considered statistically significant.

## Results

### Laboratory parameters and clinical features

**Fig. 1:**
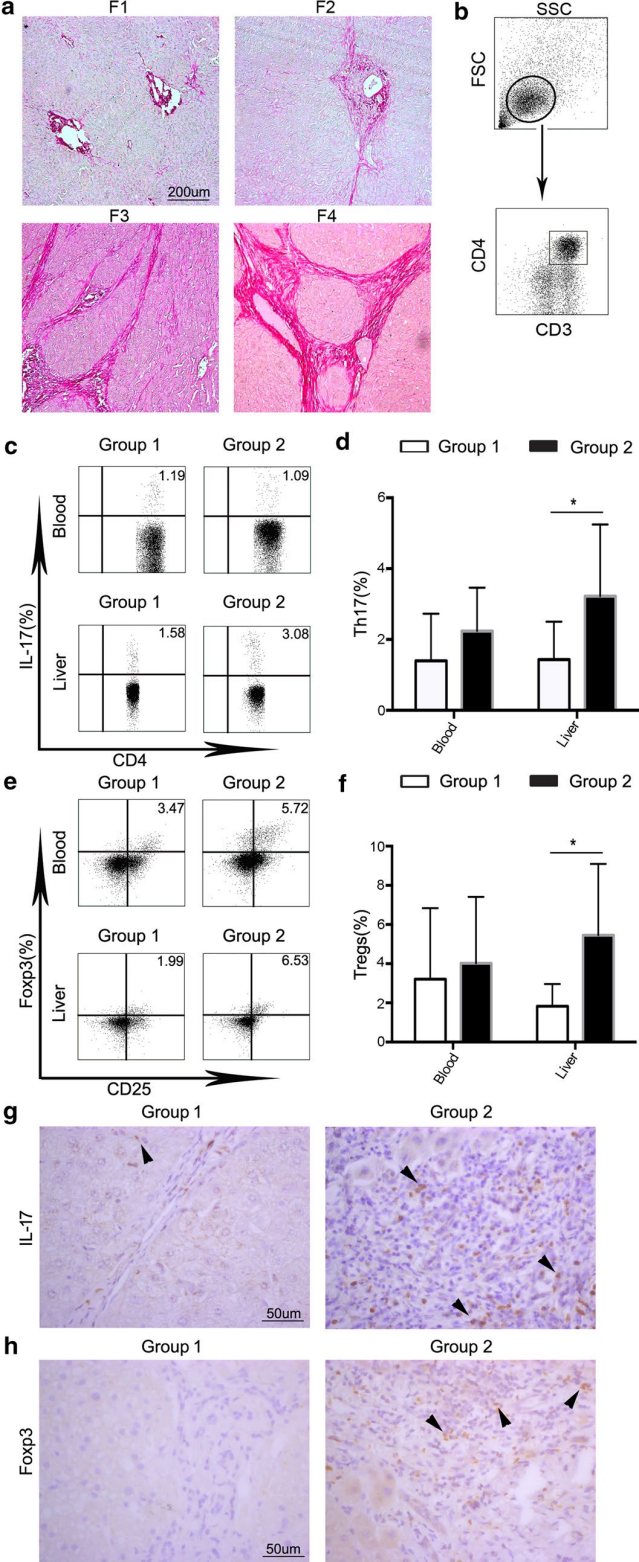
The levels of Th17 cells and Tregs in patients with different stages of HBV-LF. **a** The different stages of fibrosis were assessed by *Sirius Red* staining of liver tissues. The following scores were assigned to the different stages of fibrosis by the Laennec system: *F1* portal fibrosis without septa, *F2* portal fibrosis with rare septa, *F3* numerous septa with bridging fibrosis without cirrhosis, and *F4* cirrhosis. Patients with *F1* through *2* were classified as Group 1 and patients with *F3* or *4* were classified as Group 2. **b** CD4+ T cells gating strategy. Lymphocytes were derived from total live PBMCs/hepatic mononuclear cells gated by forward and side scatter. CD4+ T cells were defined by double positive of CD3 and CD4. **c**, **e** Flow cytometry analysis of the percentages of Th17 cells (**c**) and Tregs (**e**) in freshly isolated CD4+ T cells from peripheral blood and tissues. The values in the quadrants represent the percentage of Th17 cells and Tregs. The data shown are representative dot plots of at least 10 individuals from more than three independent experiments. **d**, **f** Comparision of the percentages of Th17 cells and Tregs between two groups. The percentages of both Th17 cells (**d**) and Tregs (**f**) increased significantly in liver tissues but not in peripheral blood in Group 2 (*black filled* profiles) compared with Group 1 (*open* profiles). **e**, **f** Liver tissues from different stages of liver fibrosis were immunostained with antibodies against IL-17 and Foxp3 in representative samples. The numbers of IL-17+ cells (**g**) and Foxp3+ cells (**h**) were significantly higher in Group 2than in Group 1. Positive cells are highlighted by *black arrow*. One of 10 representative micrographs is shown. *p < 0.05, **p < 0.01, ***p < 0.001

A total of 32 patients were enrolled in the study according to the criteria described above. The baseline characteristics are included in Table 1. Fourteen patients with fibrosis scores of 1 through 2 were classified as Group 1 (early HBV-LF), and 18 patients with fibrosis scores of 3 or 4 were classified as Group 2 (advanced HBV-LF) (Table 1). We graded the fibrosis level according to Sirius red staining of liver tissues (Fig. 1a). The distribution of patients into two groups did not reveal significant differences in the clinical parameters (Table 1). All patients were positive for HBsAg, and 31.3% of patients were HBeAg positive.

**Table 1:** Baseline characteristics of the patients included in this study

Variable	Group 1 (n = 14)	Group 2 (n = 18)	*p* value
Age, y			0.9782
Mean (SEM)	47.9 (4.161)	47.7 (2.858)	
Gender, n (%)			0.2150
Male	9 (64.3)	16 (88.9)	
Female	5 (35.7)	2 (11.1)	
ALT, U/l			0.7254
Mean (SEM)	88.6 (37.92)	107.5 (36.67)	
ALB, g/l			0.7800
Mean (SEM)	39.2 (1.176)	38.7 (0.9917)	
AFP, ng/ml			0.9835
Mean (SEM)	323.8 (132.1)	327.5 (116.9)	
TBIL, μmol/l			0.5934
Mean (SEM)	13.8 (2.043)	15.9 (2.970)	
HBeAg, n (%)			0.4110
Positive	2 (14.3)	6 (33.3)	
Negative	12 (85.7)	12 (66.7)	
HBV DNA, log_10_ IU/ml			0.6143
Mean (SEM)	4.3 (0.5422)	4.0 (0.4698)	
HBV genotype, n(%)			0.9170
B	2 (14.3)	2 (11.1)	
C	5 (35.7)	5 (27.8)	
C + D	1 (7.1)	1 (5.6)	
Missing	6 (42.9)	10 (55.5)	
Child-pugh, n(%)			1.0000
A	14 (100)	17 (94.4)	
B	0 (0)	1 (5.6)	
C	0 (0)	0 (0)	
PLT, 10^9^/l			0.1666
Mean (SEM)	204.8 (20.58)	164.8 (19.01)

### Levels of Th17 cells and CD4+ CD25+ Foxp3+ Tregs synchronously increased in advanced HBV-LF

To ascertain the role of Th17 cells and CD4+ CD25+ Foxp3+ Tregs in HBV-LF, we compared the percentages of hepatic and circulating Th17 cells and Tregs between early and advanced HBV-LF by flow cytometry. In contrast to patients with early HBV-LF, the percentages of hepatic Th17 cells and Tregs significantly elevated in patients with advanced HBV-LF (*p* = 0.0115, *p* = 0.0309, respectively) (Fig. 1b–f). They did not differ in PB between the two groups (Fig. 1b–f). Likewise, by immunohistochemistry we found that the amounts of IL-17+ T cells and Foxp3+ cells in liver tissues were augmented in advanced HBV-LF compared with early HBV-LF (Fig. 1g, h). The differences in the numbers of hepatic Th17 cells and Tregs between two groups indicated that the fibrosis environment increased the levels of Th17 cells and Tregs in situ.

### Supernatant from HSC increased the percentages of Th17 cells and Tregs

**Fig. 2:**
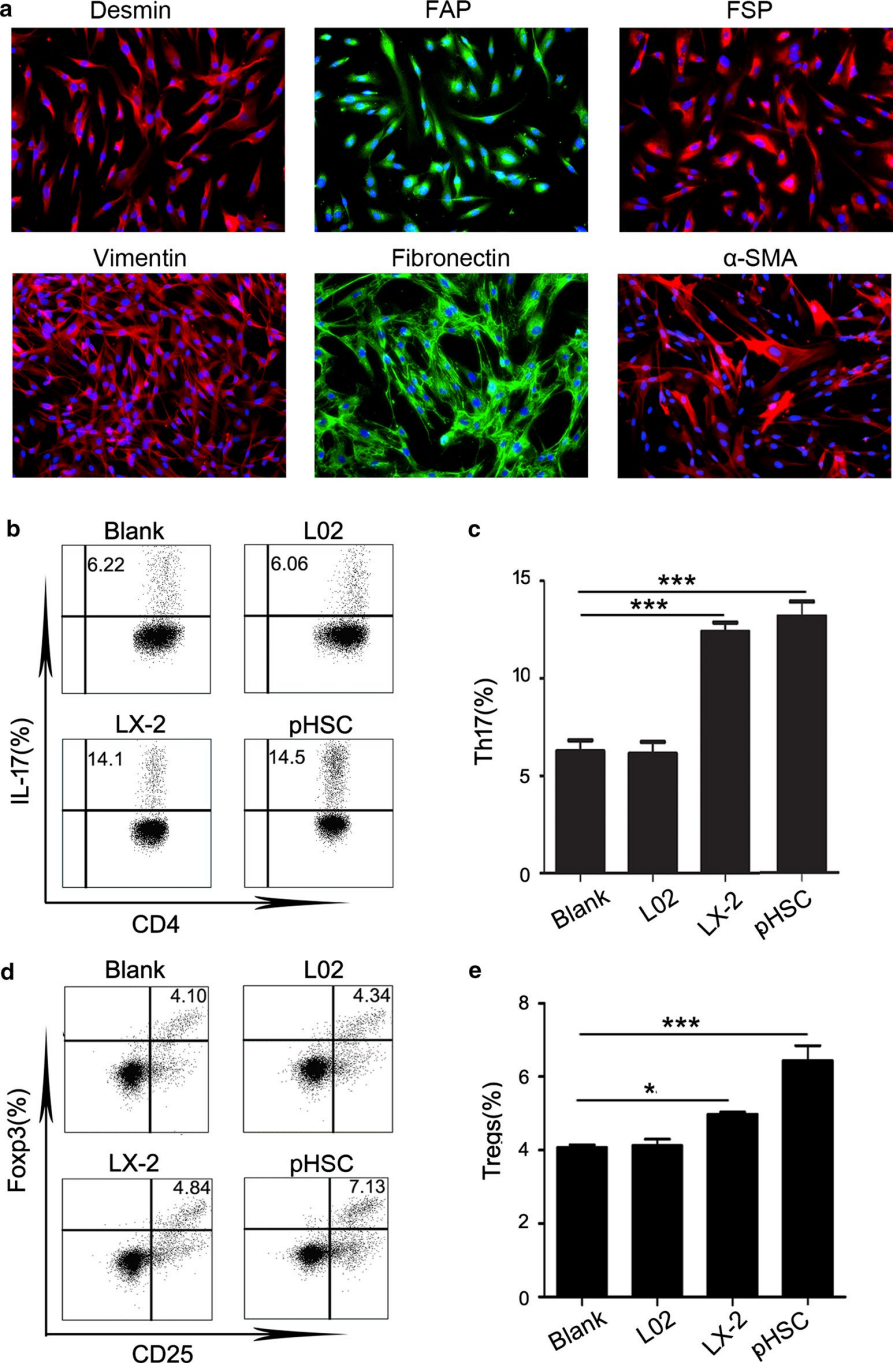
Supernatants from HSC increased the percentages of Th17 cells and Tregs. **a** Phenotypes of primary HSC extracted from HBV-related fibrotic liver tissues in Group 2. Sections were immunostained with desmin, FAP, FSP, vimentin, fibronectin and a-SMA antibodies. One of 20 representative micrographs is shown. **b**, **d** Purified CD4+ T cells were cultured alone (Blank) or with 30% indicated supernatants. The values in the quadrants represent the percentages of Th17 cells and Tregs. The data shown are representative *dot plots* from more than three independent experiments. **c**, **e** The statistical analysis of the effect of LX-2 and pHSC supernatant on the percentages of Th17 cells (**c**) and Tregs (**e**). *p < 0.05, **p < 0.01, ***p < 0.001

Studies show that HSC play a key role in the process of LF [26]. Thus, we firstly study whether HSC regulated the percentages of Th17 cells and Tregs. To this end, we extracted pHSC from HBV-related fibrotic liver tissue. The purity of pHSC was verified by fluorescence microscopy. All cell populations cultured in vitro strongly expressed fibroblast-specific markers, including desmin, FAP, FSP, vimentin, fibronectin, and α-SMA (Fig. 2a). Next, we cultured purified CD4+ T cells sorted by MACS with 30% LX-2 and pHSC supernatant for 5 days. We found that both LX-2 and pHSC supernatant increased the Th17 cells levels (Fig. 2b, c). Smilarly, our results showed that both LX-2 and pHSC supernatant increased the Tregs levels (Fig. 2d, e). Next, we wanted to know whether the supernatant from HSC increased the proliferation or differentiation of Th17 cells and Tregs. Thus, we performed the proliferation experiment by measuring the expression of ki67 on Th17 cells and Tregs under the regulation of supernatant from HSC. We found that HSC had no significant effect on the ki67 expression of both Th17 cells and Tregs (Additional file 2: Figure S1). Thus, these results indicated that HSC supernatant can actually increase the levels of Th17 cells and Tregs by promoting the differentiation of T cells.

### HSC increased the levels of Th17 cells and Tregs via the PGE2/EP2 and EP4 pathway

**Fig. 3:**
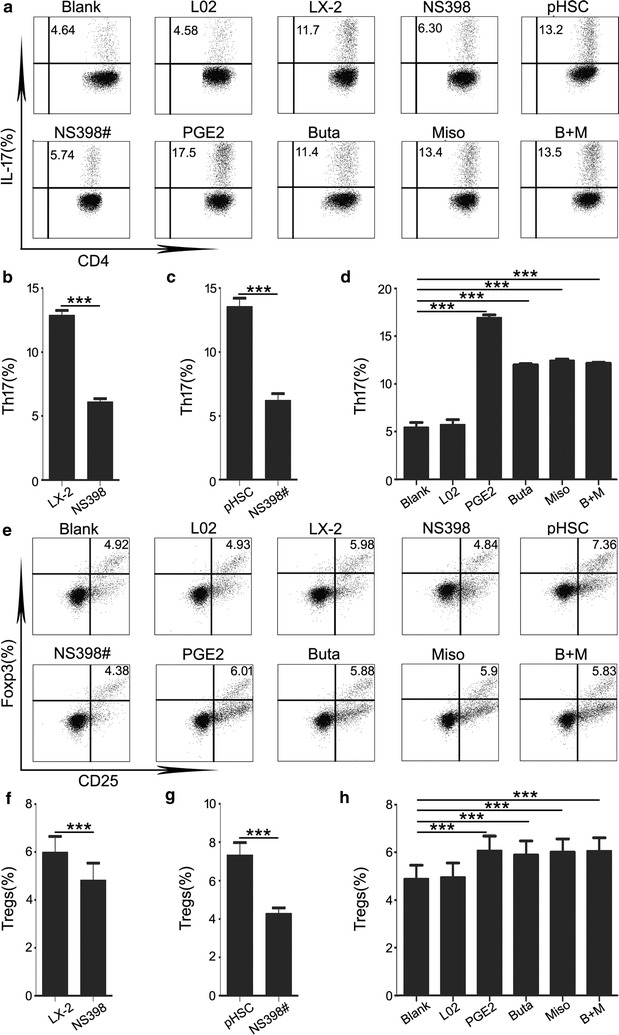
HSC increased the levels of Th17 cells and Tregs via the PGE2/EP2 and EP4 pathway. **a**, **e** Purified CD4+ T cells were cultured alone or with 30% indicated supernatants with or without NS398 or with 0.1 µM PGE2, Butaprost or Misoprostol. After 5 days, the percentage of Th17 cells and Tregs was analysed by flow cytometry. The values in the quadrants represent the percentages of Th17 cells and Tregs. The data shown are representative dot plots from more than three independent experiments. **b**, **f** The statistical analysis of the percentages of Th17 cells and Tregs in cultured purified CD4+ T cells with LX-2 supernatant or pretreated with NS398. **c**, **g** The statistical analysis of the percentages of Th17 cells and Tregs in cultured purified CD4+ T cells with pHSC supernatant or pretreated with NS398. **d**, **h** The statistical analysis of the percentages of Th17 cells and Tregs in cultured purified CD4+ T cells with PGE2, Butaprost or Misoprostol. Supernatant from LX-2 and pHSC significantly increased the percentage of Th17 cells and Tregs, and when with pretreated with NS398, the percentages of Th17 cells and Tregs declined significantly. PGE2, and its receptor agonists Butaprost and Misoprostol increased significantly the percentages of Th17 cells and Tregs. (NS398 indicates LX-2 supernatant pretreated with NS398; NS398# indicates pHSC supernatant pretreated with NS398). *p < 0.05, **p < 0.01, ***p < 0.001

It has been reported that PGE2 can not only regulate Th17 cell differentiation and function but also promote Foxp3 expression and Tregs activity through EP2/EP4 receptor signalling [27, 28]. To further ascertain if LX-2 and pHSC augmented the levels of Th17 cells and Tregs via PGE2, we cultured purified CD4+ T cells with 30% pretreated LX-2or pHSC supernatant with NS398 for 5 days. We found that the percentages of Th17 cells and Tregs cultured with pretreated LX-2 or pHSC supernatant declined significantly (Fig. 3b, c, f and g). Moreover, we found that PGE2 can enhance the levels of Th17 cells and Tregs when culturing CD4+ T cells with 0.1 µM PGE2 (Fig. 3d, h). These results revealed that both LX-2 and pHSC can increase Th17 cells and Tregs levels through PGE2.

We found that both circulating and hepatic CD4+ T cells expressed PGE2 receptors-EP2 and EP4 (Additional file 3: Figure S2). Hence, we speculated whether PGE2 acts on EP2 and EP4 to upregulate the levels of Th17 cells and Tregs. We cultured purified CD4+ T cells with the selective EP2 agonist Butaprost (0.1 µM) or the EP4 agonist Misoprostol (0.1 µM) for 5 days. We found that both Butaprost and Misoprostol enhanced the levels of Th17 cells and Tregs, and there was no further increase when combining them (Fig. 3d, h). These results illustrated that both EP2 and EP4 agonists elevate the percentages of Th17 cells and Tregs. Taken together, our results demonstrated that HSC can upregulate the levels of Th17 cells and Tregs through the PGE2/EP2 and EP4 pathway.

## Discussion

Liver fibrosis mainly occurs upon chronic hepatitis virus infection and potentially leads to portal hypertension, hepatic failure and HCC [29]. However, the relationship between the immune cells and HBV-LF has remained elusive. Here, we observed significantly higher percentages of hepatic Th17 cells and CD4+ CD25+ Foxp3+ Tregs in advanced HBV-LF. PGE2 secreted by HSC acted on its receptors EP2 and EP4 to perform this function. These data suggest a latent mechanism in which human HSC are equipped to simultaneously upregulate the levels of Th17 cells and Tregs via the PGE2/EP2 and EP4 pathway to affect the development of HBV-LF and even HCC.

Increased expression of IL-17 was detected in livers from patients with severe liver fibrosis or cirrhosis (Group 2) (Fig. 1c, d and g). Considering pro-inflammation function of IL-17 and the close relationship between cancer and inflammation [30], the increased hepatic Th17 cells in advanced HBV-LF might effectively explain the continued inflammation and HCC pathogenesis. However, the accurate molecular mechanisms underlying LF and Th17 are still poorly defined. Although some studies have reported that IL-17 is involved in the pathogenesis of LF [15, 31], few studies focus on the role of HBV-LF on Th17 cells. In our study, increased hepatic Th17 cells in advanced HBV-LF effectively revealed the immune status in hepatic microenvironment. So far, the role of IL-17 in tumourigenesis remains controversial. While some studies demonstrate that IL-17/Th17 cells promote tumor development by various ways [32, 33], others revealed that IL-17 is involved in tumor surveillance in immunocompetent mice and has anti-tumor ability in mice [34, 35]. It is necessary to ascertain whether Th17 cells predominantly skew towards pro-tumor or anti-tumor functions. Critical evaluation in appropriate mouse models of cancer using genetically altered animals lacking specific IL-17/IL-17R isoforms should address this issue.

Tregs can impair innate and adaptive immune responses by suppressing the responses of antigen presenting cells (APC), natural killer (NK) cells, NKT cells, T and B cells [36–40]. While abundant data demonstrate that there is a very close relationship between increased Tregs and tumor progression, impaired anti-tumor immunity or poor survival [21, 41, 42], the role of Tregs in the development of fibrosis remains controversial. Some studies find that Tregs can promote fibrosis. In hepatitis C, Tregs modify the interaction between NK cells and HSC in a cell-contact-dependent manner and by secreting soluble factors, resulting in the altered control of hepatic fibrogenic activity [43] and contributing to fibrogenesis via inducing the upregulation of profibrogenic markers in pHSC [18]. TGF-β produced by CD4+ Foxp3+ Tregs may worsen fibrosis by activating HSC. However, Tregs are likely to be only a minor source of free active TGF-β in the liver, and TGF-β bound to the membrane of Tregs only inhibits other immune cells in close proximity [13, 44]. IL-10 produced by Tregs can inhibit collagen matrix deposition by HSC [45], and CD4+ Foxp3+ Tregs may inhibit the effector functions of other intrahepatic T cells, thereby indirectly inhibiting HSC activation [46]. The different Tregs identification, species (human/mouse) and disease models might partly account for the different roles of Tregs in the development of fibrosis. Limited information concerns the function of hepatic Tregs in HBV-LF. In our study, we found a significant increase hepatic CD4+ CD25+ Foxp3+ Tregs in advanced HBV-LF compared to early HBV-LF. Researches have demonstrated that CD4+ CD25+ Foxp3+ Tregs inhibited proliferation, activation, degranulation, and production of granzyme A, granzyme B, and perforin of CD8+ T cells induced by anti-CD3/CD28 antibodies [21] and are potent suppressors of autologous tumor-specific T cell responses [47]. Briefly, these results demonstrates a very close relationship between Tregs and liver fibrosis, which likely accounts for the development of HBV-LF and HCC following LF considering the immune suppression function of Tregs. In the future, more studies are necessary to ascertain the exact function of Tregs, especially in the liver microenvironment.

Notably, HSC activation dominates in the process of LF and cirrhosis. Most stromal cells can produce large quantities of PGE2, and our previous study reported that HSC secrete higher concentration of PGE2 than normal skin fibroblasts [4]. In our study, we found the concentration of PGE2 in Group 2 patients increased significantly compared to Group 1 patients (Additional file 4: Figure S3). Cyclooxygenase-2(COX-2) is the rate-limiting enzyme in PGE2 synthesis. However, the role of COX-2/PGE2 in the development of LF remains ambiguous. Elevated COX-2 in fibrosis/cirrhosis suggests a potential role of COX-2/PGE2 signaling in hepatic fibrogenesis. Furthermore, many investigators have reported that COX-2/PGE2 signaling accelerates LF by promoting HSC cell line proliferation [48] and upregulating α-SMA on HSC cell lines [49]. However, a separate study showed that PGE2 inhibit collagen I synthesis in a HSC cell line [50], suggesting that PGE2 may inhibit HSC activation to limit LF. Existing studies dispute the role of PGE2 in HSC cell lines and LF. Moreover, few studies focus on the relationship among PGE2, HSC (especially pHSC) and immune cells in HBV-LF. We demonstrated that PGE2 secreted by not only LX-2 but also pHSC can simultaneously augment the percentages of Th17 cells and Tregs via its receptors EP2 and EP4, which is similar with previous reports [28, 51]. PGE2 has both pro- and anti-proliferative effects depending on cell type [52, 53], pro- and anti-inflammatory effects depending on the context and target of its action [54, 55], and pro- and anti-apoptotic effects depending on the maturation and activation state of T cells [43, 48]. Our data show that PGE2 secreted by HSC act on EP2 and EP4 to increase synchronously the levels of Th17 cells and Tregs, providing novel support for the dual function of PGE2 from the perspective of inflammatory response and immune suppression and potentially accounting for the continuous LF.

Some data show that IL-17/Th17 cells and Tregs can induce HSC activation to accelerate the pathogenesis of LF [13, 14, 31, 44, 50]. In turn, in our study, these activated HSC upregulate the levels of Th17 cells and Tregs via the PGE2/EP2 and EP4 pathway. This pathway forms a detrimental loop, leading to the deterioration of LF and the pathogenesis of HCC probably. Furthermore, the Th17/Tregs balance is important in inflammatory diseases and autoimmune diseases. In the current study, both hepatic Th17 cells and Tregs were increased in advanced HBV-LF, which is similar to existing studies in the tumor environment [56]. This imbalance further contributes to the development of LF, cirrhosis and even HCC.

There are some limitations in our research. The isolation of “functional” Tregs in vitro is difficult because traditional Tregs isolation depends on CD25 expression, but CD25 can be expressed on activated T cells upon activation. Thus, it is difficult to study the phenotype and function of “functional” Tregs in vitro. In addition, studies targeting the intrahepatic lymphocytes are rare, especially in fibrotic liver. Therefore, the presence and action of Th17 cells and Tregs in the liver, the site of virus replication and chronic inflammation, should be analyzed specifically.

## Conclusions

HSC promoted the differentiation of hepatic Th17 cells and CD4+ CD25+ Foxp3+ Tregs in advanced HBV-LF patients by secreting PGE2. These results provide a novel mechanism of continuous inflammation and liver fibrosis. Based on these observations, we suggest that potent immunotherapy targeting Th17 cells and CD4+ CD25+ Foxp3+ Tregs and the inhibition of PGE2 secretion from HSC can slow the progress of LF and cirrhosis and other associated complications.

## Additional files

**Additional file 1.** Additional material.

**Additional file 2: Figure S1.** Expression of ki67 on Th17 cells and Tregs regulated by HSC. (A, C) the expression of ki67 on Th17 cells (A) and Tregs (C) regulated by LX-2 cell lines. (B, D) the expression of ki67 on Th17 cells (B) and Tregs (D) regulated by pHSC. Both LX-2 and pHSC did not affect significantly the expression of ki67 on Th17 cells and Tregs.

**Additional file 3: Figure S2.** The expression of EP2 and EP4.Flow cytometry analysis of the expression of EP2 and EP4 in freshly isolated CD4+ T cells from peripheral blood and liver tissues. The histogram indicates that both circulating and intrahepatic CD4+ T cells express EP2 and EP4 receptors (black line: isotype, grey line: EP2 or EP4). The data shown are representative histograms of at least 10 individuals from more than three independent experiments.

**Additional file 4: Figure S3.** Concentration of serum PGE2 of patients. Statistic analysis of the concentration of serum PGE2 in Group 2 (black filled profiles) compared with Group 1 (open profiles) by ELISA. *: p < 0.05, **: p < 0.01, ***: p < 0.001.

## Abbreviations

HBV: hepatitis B virus
HBV-LF: HBV-related liver fibrosis
HCC: hepatocellular carcinoma
HSC: hepatic stellate cell
Th17 cells: IL-17-producing CD4+ T cells
Tregs: regulatory T cells
PB: peripheral blood
FSP: fibroblast surface protein
a-SMA: alpha smooth muscle actin
FAP: fibroblast activation protein
MACS: magnetic cell sorting
PGE2: prostaglandin E2
Foxp3: forkhead box protein 3
APC: antigen presenting cells
NK: natural killer
COX-2: cyclooxygenase-2
