# Supplementary material for: Levels of hepatic Th17 cells and regulatory T cells upregulated by hepatic stellate cells in advanced HBV-related liver fibrosis
Source: J Transl Med. 2017 Apr 11;15:75. doi: 10.1186/s12967-017-1167-y (PMC5387242; doi:10.1186/s12967-017-1167-y)
Supplement: Supplementary file 1 — Additional file 1. Additional material. [file 12967_2017_1167_MOESM1_ESM.docx]

Supplement Figure 4. The expression of Zombie Aqua. The total cells were lymphocytes gated by **f**orward and side scatter characteristics.

**
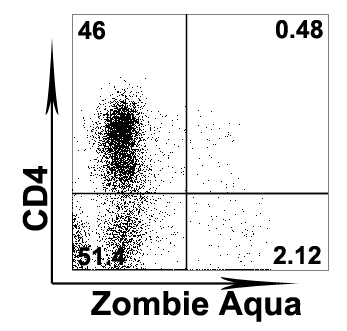
**
