# Supplementary figures and images for: Levels of hepatic Th17 cells and regulatory T cells upregulated by hepatic stellate cells in advanced HBV-related liver fibrosis
Source: J Transl Med. 2017 Apr 11;15:75. doi: 10.1186/s12967-017-1167-y (PMC5387242; doi:10.1186/s12967-017-1167-y)

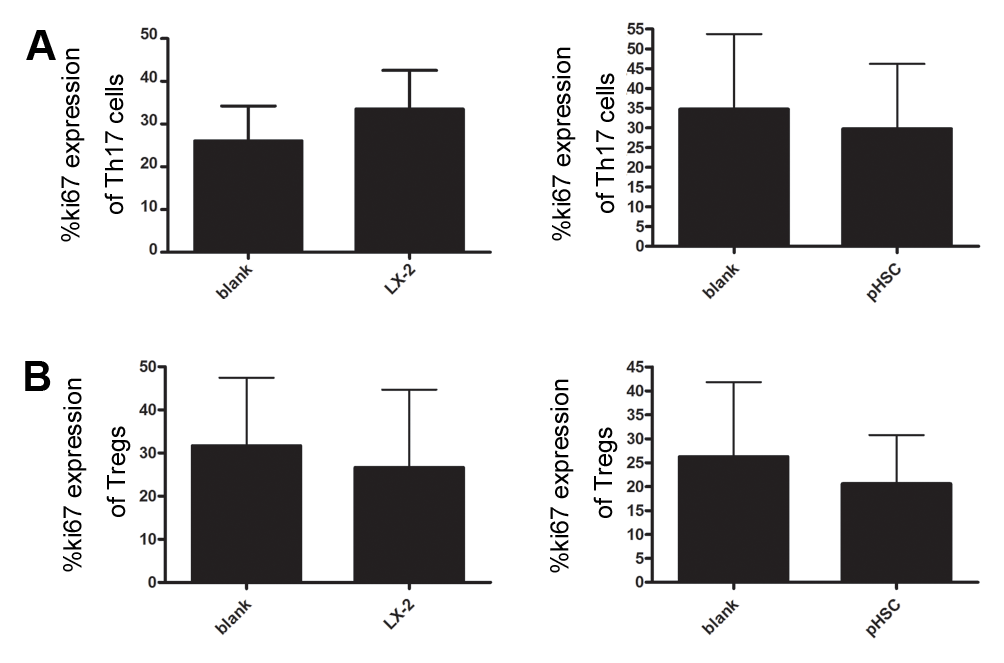

Supplement: Supplementary file 2 — Additional file 2: Figure S1. Expression of ki67 on Th17 cells and Tregs regulated by HSC. (A, C) the expression of ki67 on Th17 cells (A) and Tregs (C) regulated by LX-2 cell lines. (B, D) the expression of ki67 on Th17 cells (B) and Tregs (D) regulated by pHSC. Both LX-2 and pHSC did not affect significantly the expression of ki67 on Th17 cells and Tregs. [file 12967_2017_1167_MOESM2_ESM.tif]

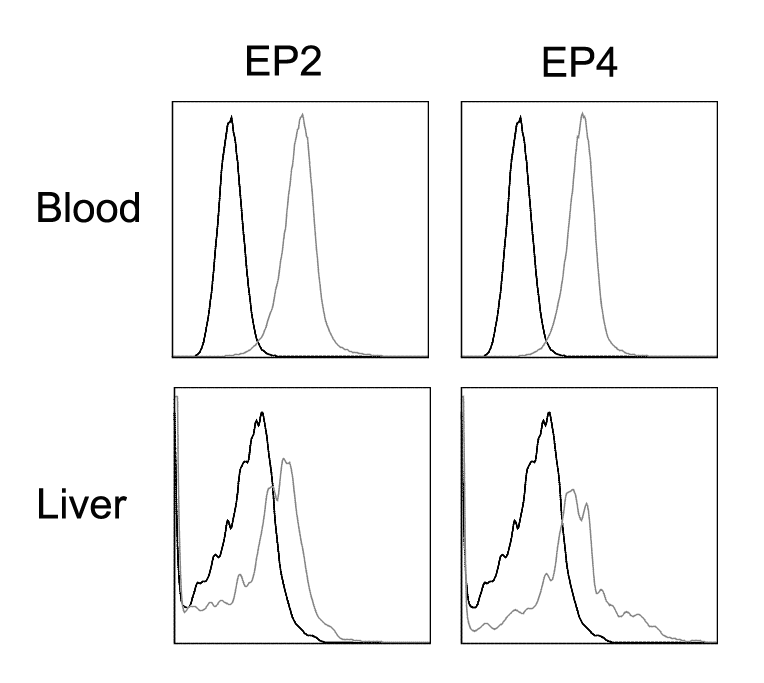

Supplement: Supplementary file 3 — Additional file 3: Figure S2. The expression of EP2 and EP4.Flow cytometry analysis of the expression of EP2 and EP4 in freshly isolated CD4+ T cells from peripheral blood and liver tissues. The histogram indicates that both circulating and intrahepatic CD4+ T cells express EP2 and EP4 receptors (black line: isotype, grey line: EP2 or EP4). The data shown are representative histograms of at least 10 individuals from more than three independent experiments. [file 12967_2017_1167_MOESM3_ESM.tif]

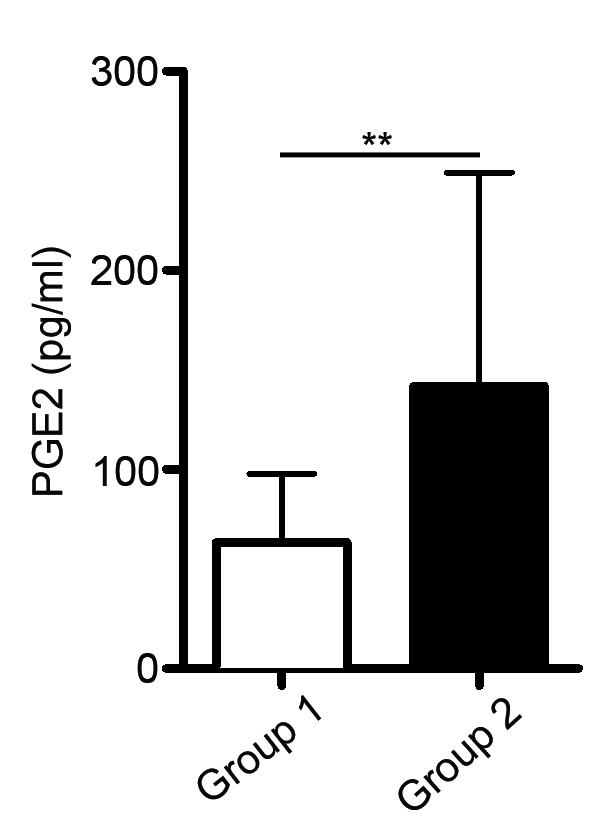

Supplement: Supplementary file 4 — Additional file 4: Figure S3. Concentration of serum PGE2 of patients. Statistic analysis of the concentration of serum PGE2 in Group 2 (black filled profiles) compared with Group 1 (open profiles) by ELISA. *: p < 0.05, **: p < 0.01, ***: p < 0.001. [file 12967_2017_1167_MOESM4_ESM.tif]
